# Changes in the salivary metabolome in patients with chronic erosive gastritis

**DOI:** 10.1186/s12876-023-02803-6

**Published:** 2023-05-19

**Authors:** Shaowei Liu, Shixiong Zhang, Haoyu Chen, Pingping Zhou, Tianxiao Yang, Jingjing Lv, Huixia Li, Yangang Wang

**Affiliations:** 1 Hebei University of Chinese Medicine, Xinshi South Road No 326, Qiaoxi District, Shijiazhuang, Hebei 050091 China; 2 Nanjing University of Chinese Medicine, 138 Xianlin Avenue, Nanjing, Jiangsu 210023 China; 3 Hebei Hospital of Traditional Chinese Medicine, Zhongshan East Road No 389, Changan District, Shijiazhuang, Hebei 050011 China; 4 Beijing University of Chinese Medicine Third Affiliated Hospital, Anwai Xiaoguan Street No. 51, Chaoyang District, Beijing, 100029 China

**Keywords:** Chronic erosive gastritis, Saliva, Metabolomics, Biomarker, UHPLC-Q-TOF/MS

## Abstract

**Introduction:**

Chronic erosive gastritis (CEG) is closely related to gastric cancer, which requires early diagnosis and intervention. The invasiveness and discomfort of electronic gastroscope have limited its application in the large-scale screening of CEG. Therefore, a simple and noninvasive screening method is needed in the clinic.

**Objectives:**

The aim of this study is to screen potential biomarkers that can identify diseases from the saliva samples of CEG patients using metabolomics.

**Methods:**

Saliva samples from 64 CEG patients and 30 healthy volunteers were collected, and metabolomic analysis was performed using UHPLC-Q-TOF/MS in the positive and negative ion modes. Statistical analysis was performed using both univariate (Student’s t-test) and multivariate (orthogonal partial least squares discriminant analysis) tests. Receiver operating characteristic (ROC) analysis was conducted to determine significant predictors in the saliva of CEG patients.

**Results:**

By comparing the saliva samples from CEG patients and healthy volunteers, 45 differentially expressed metabolites were identified, of which 37 were up-regulated and 8 were down-regulated. These differential metabolites were related to amino acid, lipid, phenylalanine metabolism, protein digestion and absorption, and mTOR signaling pathway. In the ROC analysis, the AUC values of 7 metabolites were greater than 0.8, among which the AUC values of 1,2-dioleoyl-sn-glycoro-3-phosphodylcholine and 1-stearoyl-2-oleoyl-sn-glycoro-3-phospholine (SOPC) were greater than 0.9.

**Conclusions:**

In summary, a total of 45 metabolites were identified in the saliva of CEG patients. Among them, 1,2-dioleoyl-sn-glycoro-3-phosphorylcholine and 1-stearoyl-2-oleoyl-sn-glycoro-3-phosphorine (SOPC) might have potential clinical application value.

**Supplementary Information:**

The online version contains supplementary material available at 10.1186/s12876-023-02803-6.

## Introduction

Chronic erosive gastritis (CEG) is a kind of gastritis characterized by impaired integrity of gastric mucosa. It shows flat or uplift erosion under a gastroscope. The depth of wound rupture is no more than 1 mm and does not reach the muscular layer [1]. Clinically, CEG can be manifested as stomach pain, nausea, vomiting, anorexia, weight loss and other symptoms. In severe cases, anemia may be caused by bleeding, but many patients do not have any symptoms [2]. Long-term use of NSAIDs is the most common cause of CEG. In addition, *Helicobacter pylori* infection, bile reflux, alcohol, cocaine and ionizing radiation are also the factors causing CEG [3, 4]. The morbidity of CEG is high. A multicenter study on chronic gastritis in China showed that 3760 (42.3%) of 8892 patients were diagnosed as CEG under an electronic gastroscope [5]. Long-term chronic inflammation of gastric mucosa is closely related to gastric cancer [6]. Therefore, early diagnosis and intervention of CEG are of great significance to delay the progression of chronic gastritis and prevent gastric cancer. At present, the diagnosis of CEG depends on gastroscopy, but it is difficult to be used as a broad screening method because of its invasiveness and discomfort [7]. Consequently, a new detection method with small trauma, easy operation and low cost is needed in the clinic.

Metabonomics is a comprehensive and effective method to analyze the changes in endogenous small molecule metabolites [8]. LC-MS-based metabonomics plays vital roles in biomarker identification and clinical diagnosis [9]. Saliva is one of the most important body fluids in human body and has many functions. Due to the physiological characteristics of saliva and salivary glands, biomarkers in blood circulation can be finally secreted into saliva [10]. Because of its low-cost, non-invasiveness, and safety, saliva collection is expected to become an alternative method of serum or urine detection, which has a good prospect for clinical diagnosis [11]. At present, saliva-based metabonomic approach has been applied to the diagnosis of diabetes [12], cardiovascular diseases [13] and various cancers [14–16], including gastric cancer [17, 18].

In the present study, a total of 94 participants were recruited, including 30 healthy volunteers and 64 CEG patients. LC-MS-based metabolomic approach was used to assess the changes in salivary metabolites between CEG patients and healthy controls.

## Methods

### Participants

Participants were recruited at the Department of Gastroenterology of Hebei Hospital of traditional Chinese medicine from September 2021 to June 2022, including 30 healthy controls (Normal group) and 64 CEG patients (CEG group). Written informed consent was obtained from all participants. This study was approved by the ethics committee of Hebei Hospital of traditional Chinese medicine.

All participants met the diagnostic criteria of CEG. The inclusion criteria were as follows: (1) diagnosed as CEG by endoscopy; (2) 25–70 years old; and (3) willingness to participate in the test and undersign the written informed consent. The exclusion criteria were as follows: (1) past or present use of NSAIDs or other agents that can cause CEG; (2) suffering from any other digestive system diseases; (3) any types of cardiovascular diseases; (4) illness of the hematological system; (5) mental disorder including depression; and (6) incapability or limited capability.

### Sample collection and preparation

All subjects were asked to avoid oral hygiene measures (such as brushing and flossing), eating, drinking, smoking, gum chewing or taking exercise 12 h before sample collection. Saliva was dynamically collected. The saliva collection took place in a quiet room from 9:00 a.m. to 11:00 a.m. Before sampling, participants should rinse their mouth with distilled water for 3–5 times to remove impurities in their mouth. After splitting out the water, an appropriate amount of saliva naturally flowing out in a quiet state was collected into a sterile sputum cup. The collected saliva samples were stored in an ice box and immediately centrifuged at 13,200 g for 10 min at 4℃. The centrifuged supernatant was sub packed and stored at -80℃ until further use.

After thawing the frozen saliva samples at 4℃, After thawing frozen saliva samples at 4℃, 100 µL saliva was taken and mixed with a cold mixture of 10µL internal standard and 300µL methanol/acetonitrile/H2O (2:2:1, v/v/v). After vortex mixing, the samples were exposed to low-temperature ultrasound for 30 min, incubated at -20℃ for 10 min, and centrifuged at 14,000 g for 20 min at 4℃. The supernatant was collected, vacuum dried, and re-dissolved with 50 µL acetonitrile aqueous solution (acetonitrile: water = 1:1, v/v). After vortexing and centrifugation at 14,000 g for 15 min at 4℃, the supernatant was injected for mass spectrometry analysis [19].

### Quality control

Quality control (QC) samples were prepared using the mixed saliva samples. Before loading the samples and throughout the whole experiment, random sequences were used for continuous analysis of the samples. QC samples were inserted into the sample queue to evaluate the stability of the system and the reliability of the experimental data.

### LC-MS/MS analysis

Saliva analyses were performed using an ultra-high performance liquid chromatography (UHPLC, 1290 Infinity LC, Agilent Technologies) coupled to a quadrupole time-of-flight mass spectrometer (AB Sciex TripleTOF 6600, Shanghai Applied Protein Technology Co., Ltd). For HILIC separation, the samples were analyzed using a 2.1 mm × 100 mm ACQUIY UPLC BEH 1.7 μm column (Waters, Ireland). In both ESI positive and negative modes, the mobile phase contained A = 25 mM ammonium acetate and 25 mM ammonium hydroxide in water and B = acetonitrile. The gradient was 85% B for 1 min, linearly reduced to 65% within 11 min, further reduced to 40% within 0.1 min, kept for 4 min, and finally increased to 85% within 0.1 min, followed by a 5-min of re-equilibration period.

For RPLC separation, a 2.1 mm × 100 mm ACQUIY UPLC HSS T3 1.8 μm column (Waters, Ireland) was used. In ESI positive mode, the mobile phase contained A = water with 0.1% formic acid and B = acetonitrile with 0.1% formic acid; and in ESI negative mode, the mobile phase contained A = 0.5 mM ammonium fluoride in water and B = acetonitrile. The gradient was 1% B for 1.5 min, linearly increased to 99% within 11.5 min, and kept for 3.5 min. Then, it was reduced to 1% within 0.1 min, followed by a 3.4 min of re-equilibration period. The gradients were maintained at a flow rate of 0.3 mL/min, and the column temperatures were kept constant at 25℃. A 2-µL aliquot of each sample was injected.

The ESI source conditions were set as follows: Ion Source Gas 1 (Gas 1) = 60, Ion Source Gas 2 (Gas 2) = 60, curtain gas (CUR) = 30, source temperature = 600℃, IonSpray Voltage Floating (ISVF) = ± 5500 V. In MS only acquisition, the instrument was set to acquire over the m/z range of 60-1000 Da, and the accumulation time for TOF MS scanning was set at 0.20 s/spectra. In auto MS/MS acquisition, the instrument was set to acquire over the m/z range of 25-1000 Da, and the accumulation time for production scanning was set at 0.05 s/spectra. The product ion scan was acquired using the information dependent acquisition (IDA) in high sensitivity mode. The parameters were set as follows: the collision energy (CE) was fixed at 35 V with ± 15 eV, declustering potential (DP) = 60 V (+) and − 60 V (−), exclude isotopes within 4 Da, and candidate ions to monitor per cycle = 10 [20].

### Raw data processing and statistical analysis

The raw MS data (wiff.scan files) were converted to MzXML files using MSConvert (ProteoWizard) before importing into freely available XCMS package (R software 4.1.3). For peak picking, the following parameters were used: centWave m/z = 25 ppm, peak width = c (10, 60), and prefilter = c (10, 100). For peak grouping, bw = 5, mzwid = 0.025, and minfrac = 0.5 were used. Collection of Algorithms of MEtabolite pRofile Annotation (CAMERA) was used for annotation of isotopes and adducts. For the extraction of ion features, only the variables with more than 50% of the nonzero measurement values in at least one group were retained. Compound identification of metabolites was performed by comparing the accuracy of m/z value (< 25 ppm), and MS/MS spectra with an in-house database were established with the corresponding authentic standards.

After normalization to total peak intensity, the processed data were uploaded before importing into SIMCA-P (version 16.1, Umetrics, Umea, Sweden), and then subjected to multivariate data analysis, including Pareto-scaled principal component analysis (PCA) and orthogonal partial least-squares discriminant analysis (OPLS-DA). The 7-fold cross-validation and response permutation testing were used to evaluate the robustness of the model. The variable importance in the projection (VIP) value of each variable in the OPLS-DA model was calculated, in order to determine its contribution to the classification.

The potential biomarkers were putatively identified by HMDB (http://www.hmdb.ca/) and METLIN (http://metlin.scripps.edu/) databases. The m/z values of the differential compounds were imported into the online databases and the tolerance was set below 10 ppm. Then, the possible compounds were obtained from the databases. The real MS/MS of differential compounds from the peak view 2.0 were matched with the MS/MS in the online databases to identify the potential biomarkers. The processed data set was imported into SPSS Statistics 21 software (IBM, USA) for Mann-Whitney test, which were corrected by false discovery rate (FDR) program. Compounds that satisfy both *P* < 0.05 and VIP > 1 were considered as differential compounds.

To evaluate the diagnostic power of potential biomarkers, receiver operator characteristic (ROC) curve analysis was performed using the SPSS Statistics 21 software. The metabolites were blasted against the online Kyoto Encyclopedia of Genes and Genomes (KEGG) database (http://www.kegg.jp/) to retrieve their COs and were subsequently mapped to pathways in KEGG11. The corresponding KEGG pathways were extracted [21–23].

## Results

### Characteristics of the study population

A total of 64 patients were included in this study. There were 31 females and 33 males, with an average age of 49.64 ± 6.80 years old. Among the 30 participants in normal group, there were 14 males and 16 females, with an average age of 47.67 ± 9.88 years old.

The results of electronic gastroscopy showed that the mucosa at the gastric body or antrum was in sheet or strip erosion, local mucosa was congested, and old bleeding spots could also be observed in CEG patients (Supplementary Fig. 1).

As shown in Table 1, there were no significant differences in gender, age, ALT, AST, ALP between the CEG and normal groups. Epigastric pain, gastric distension and heartburn were common clinical symptoms of CEG patients, but there was no significant difference in the frequency.

**Table 1:** Baseline characteristics of patients included in the present study

	CEG group(n = 64)	N group (n = 30)	P-value
Age, years	49.64 ± 6.80	47.67 ± 9.88	0.174
Male, n (%)	33 (51.56)	14 (46.67)	0.873
*Symptoms*			
Epigastric pain n (%)	34 (53.13)	0	0.000
Gastric distention n (%)	28 (43.75)	0	0.000
Heartburn n (%)	31 (48.44)	0	0.000
*Hematological results*			
ALT (U/L)	20.36 ± 6.95	28.73 ± 9.09	0.166
AST (U/L)	20.41 ± 3.75	19.77 ± 3.02	0.583
ALP (U/L)	78.32 ± 18.14	81.35 ± 11.18	0.554

### Validation of LC-MS method

**Fig. 1:**
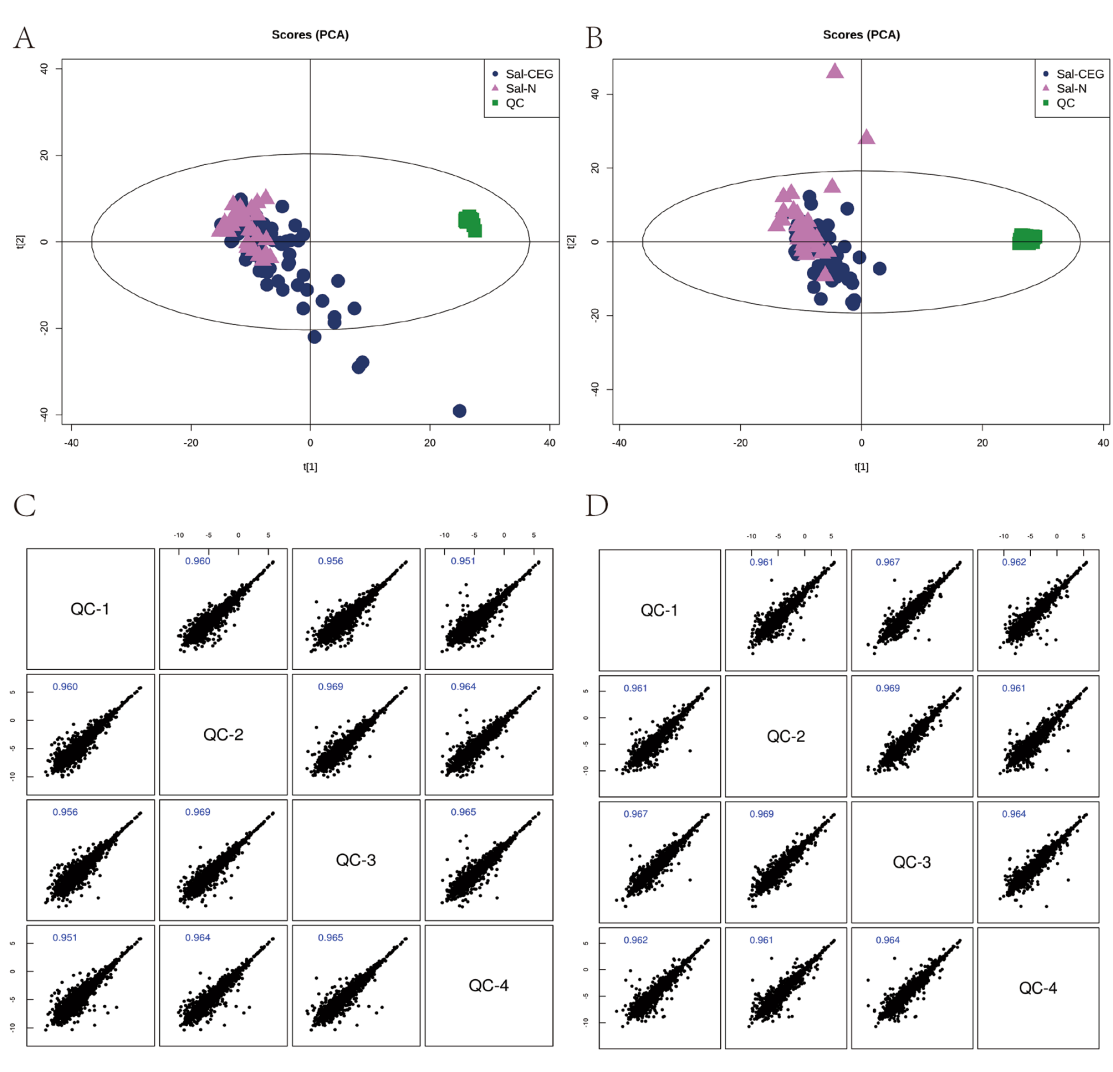
Quality control (QC) chart for samples. **A**, **B** Principal component analysis (PCA) score charts in the positive and negative ion modes, different graphs represent different samples. CEG group samples = Dark blue dots, N group samples = Purple triangles, and QC samples = Green squares. **C**, **D** QC sample correlation map for the positive and negative ion modes

To ensure the reliability of data, various methods were adopted for quality control. The results of PCA model were obtained through the pareto-scaling conversion of each peak. As shown in Fig. 1a and b, QC samples were closely clustered under the positive and negative ion modes, indicating that the repeatability of the experiment is good. Pearson correlation analysis was then performed on QC samples. The abscissa and ordinate represent the logarithm of the ion peak signal intensity value. As shown in Fig. 1c and d, the correlation coefficient between all QC samples was above 0.9, indicating that the correlation is good and the analytical system is stable [20, 24]. In addition, total ion chromatogram (TIC), multivariate control chart, relative standard deviation (RSD) of QC and Hotelling’s T2 test of each sample were also carried out (Supplementary Figs. 2 and 3). The results demonstrate that the data of this experiment is reliable and can be used for subsequent analysis.

### Multivariate statistical analysis

**Fig. 2:**
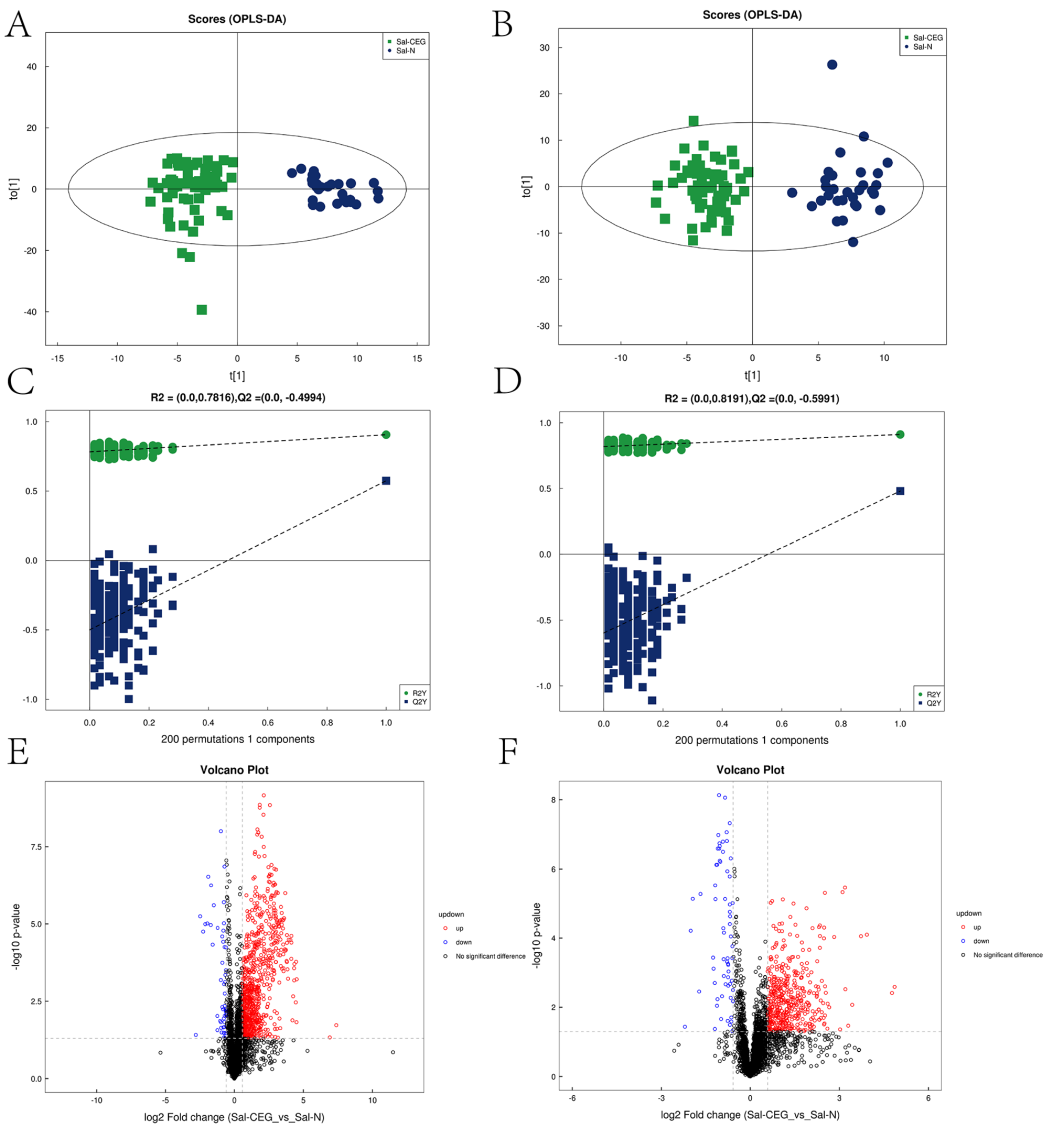
Orthogonal partial least square-discriminate analysis (OPLS-DA) score charts and cross-validation test in the positive and negative ion mode (**A**-**D**). **A**, **C** OPLS-DA score graph and mode cross-validation graph for the CEG and N groups in the positive ion mode. **B**, **D** OPLS-DA score graph and mode cross-validation graph for the CEG and N groups in the negative ion mode. CEG = Green square and N = Dark blue dots. Volcano map based on fold change (FC) analysis and t test (**E**-**F**). **E**, **F** CEG and N volcano diagrams in the positive and negative ion modes. Up regulated metabolites = Red circle, Down regulated metabolites = Blue circle

To clearly reveal the relationship between each group of samples, PCA was conducted, and there was a weak separation trend between the CEG and normal groups. Therefore, it is necessary to adjust the model to show more apparent inter group differences. OPLS-DA model was further used to distinguish the two groups of samples. As shown in the OPLS-DA score plot (Fig. 2a, b), the CEG and normal groups could be significantly separated in both positive and negative ion modes, indicating that the metabolites in the saliva of CEG patients have changed significantly compared with the healthy controls. The evaluation parameters Q^2^Y and R^2^ of OPLS-DA model were obtained through cross-validation. R2y = 0.905 and Q2 = 0.574 in the positive ionization model, R2y = 0.911 and Q2 = 0.481 in the negative ionization model, suggesting acceptable applicability and predictability. To ensure the effectiveness of the model, the permutation test was used to verify the model, and the results showed that the model was valid without overfitting (Fig. 2c, d). Next, volcano plot was used to display the results of fold change (FC) analysis and Mann-Whitney test between the two groups. As shown in Fig. 2e, f, both CEG and normal groups were significantly separated in the positive and negative ion modes.

### Screening potential biomarkers related to CEG

**Fig. 3:**
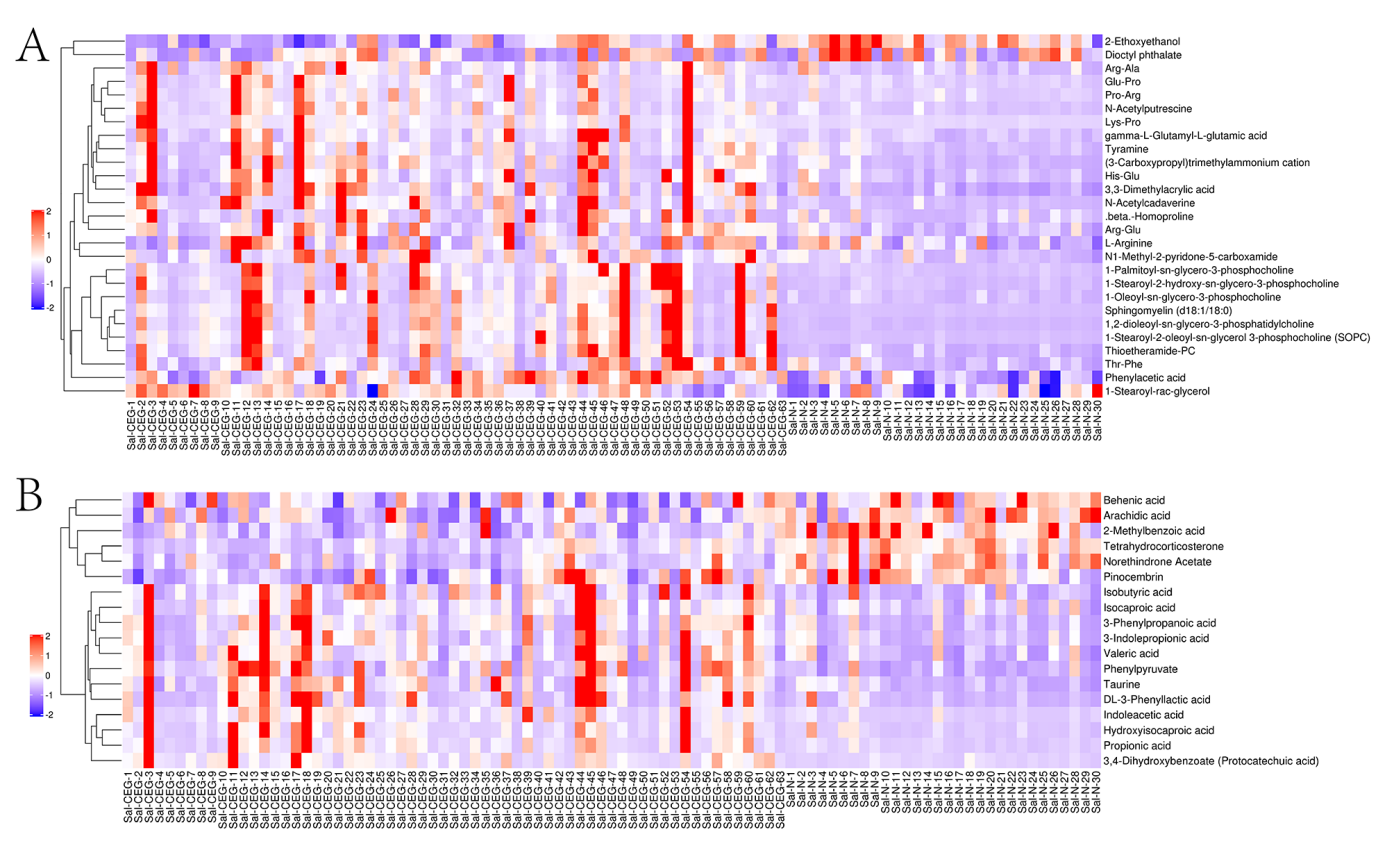
Differential metabolite hierarchical clustering diagram in positive(**A**) and negative(**B**) ion mode. The ordinate represents the metabolites that are significantly differently expressed, and the abscissa is the sample information. Red represents significantly up-regulated metabolites, blue represents significantly down-regulated metabolites, and the gray part represents no quantitative information on the metabolite

Based on the VIP obtained by OPLS-DA model and significant *p*-value obtained from the Student t-test, the differential metabolites of CEG and normal groups were screened to obtain the potential biomarkers with VIP > 1 and *p-*value < 0.05. In the positive and negative ion modes, 45 potential biomarkers were successfully identified (Supplementary Table 1). To more comprehensively and intuitively analyze the relationship between samples and the differences in metabolite expression patterns between the two groups, and evaluate the changes in related metabolic processes, the metabolite expression levels were used to cluster each group of samples. As shown in Fig. 3, compared with the normal group, the metabolites of CEG patients changed significantly. The changes in these metabolites may be associated with the pathogenesis of CEG.

**Fig. 4:**
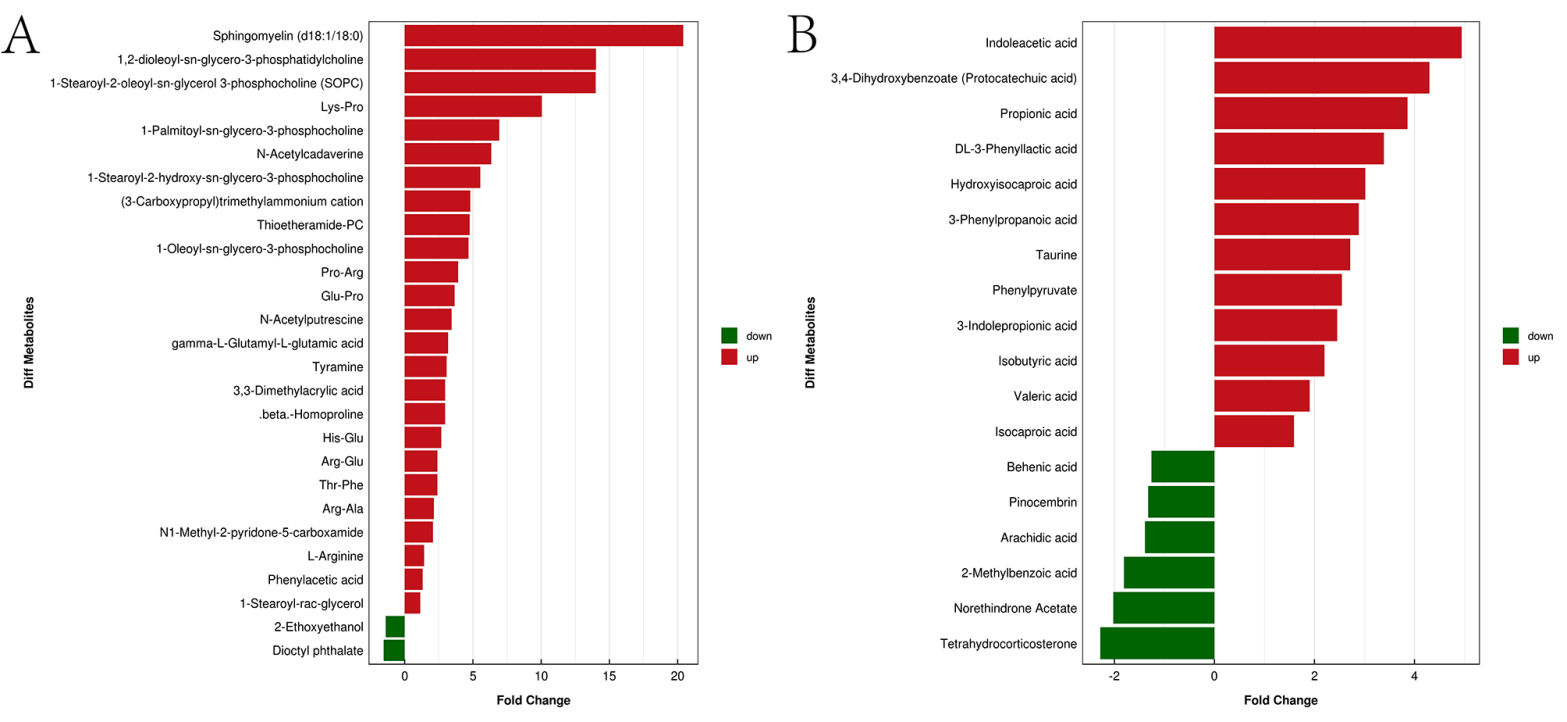
Volcano map based on fold change (FC) analysis in positive(**A**) and negative(**B**) ion mode. Up regulated metabolites = Red bar, Down regulated metabolites = Green bar

**Fig. 5:**
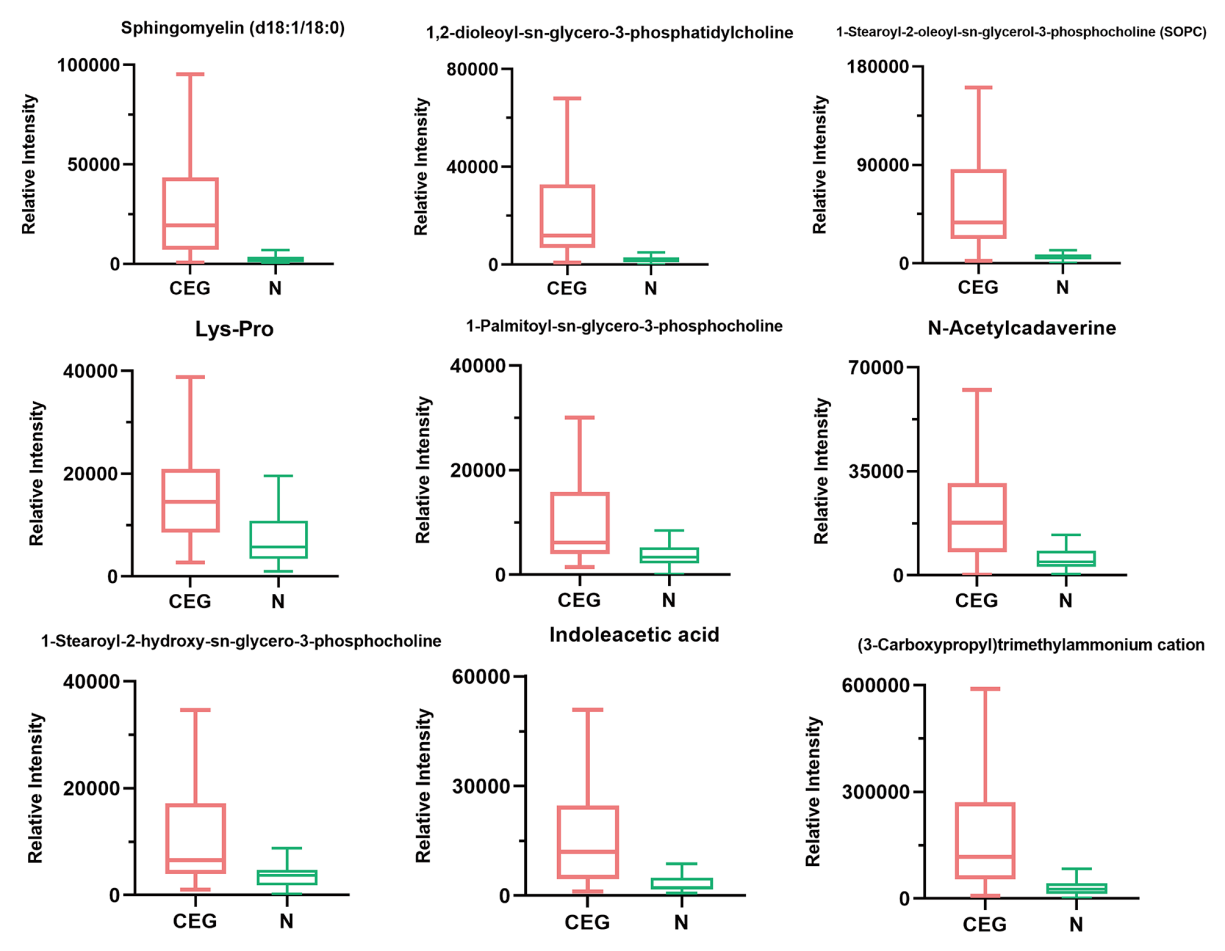
Box plots of top nine representative biomarkers screened according to their FC (Fold Change) values

To further demonstrate the change trend and amplitude of metabolites, FC plot was constructed. As shown in Fig. 4, compared with the normal group, the expression of 37 metabolites was up-regulated in the CGE group, including sphingomyelin (d18:1/18:0), 1,2-dioleoyl-sn-glycero-3-phosphatidylcholine, 1-stearoyl-2-oleoyl-sn-glycerol 3-phosphocholine (SOPC), Lys-Pro, 1-palmitoyl-sn-glycero-3-phosphocholine, N-acetylcadaverine, 1-stearoyl-2-hydroxy-sn-glycero-3-phosphocholine,indoleacetic acid, (3-carboxypropyl) trimethylammonium cation, and thioetheramide-PC. It was also observed that the expression levels of 8 metabolites such as tetrahydrocorticosterone, norethindrone acetate, 2-methylbenzoic acid, dioctyl phthalate, 2-ethoxyethanol, arachidic acid, pinocembrin, and behenic acid were down-regulated. In addition, the metabolites were ranked according to their FC values, and the box plot of top 9 representative up-regulated or down-regulated metabolites was drawn (Fig. 5).

### ROC analysis of the potential biomarkers

**Fig. 6:**
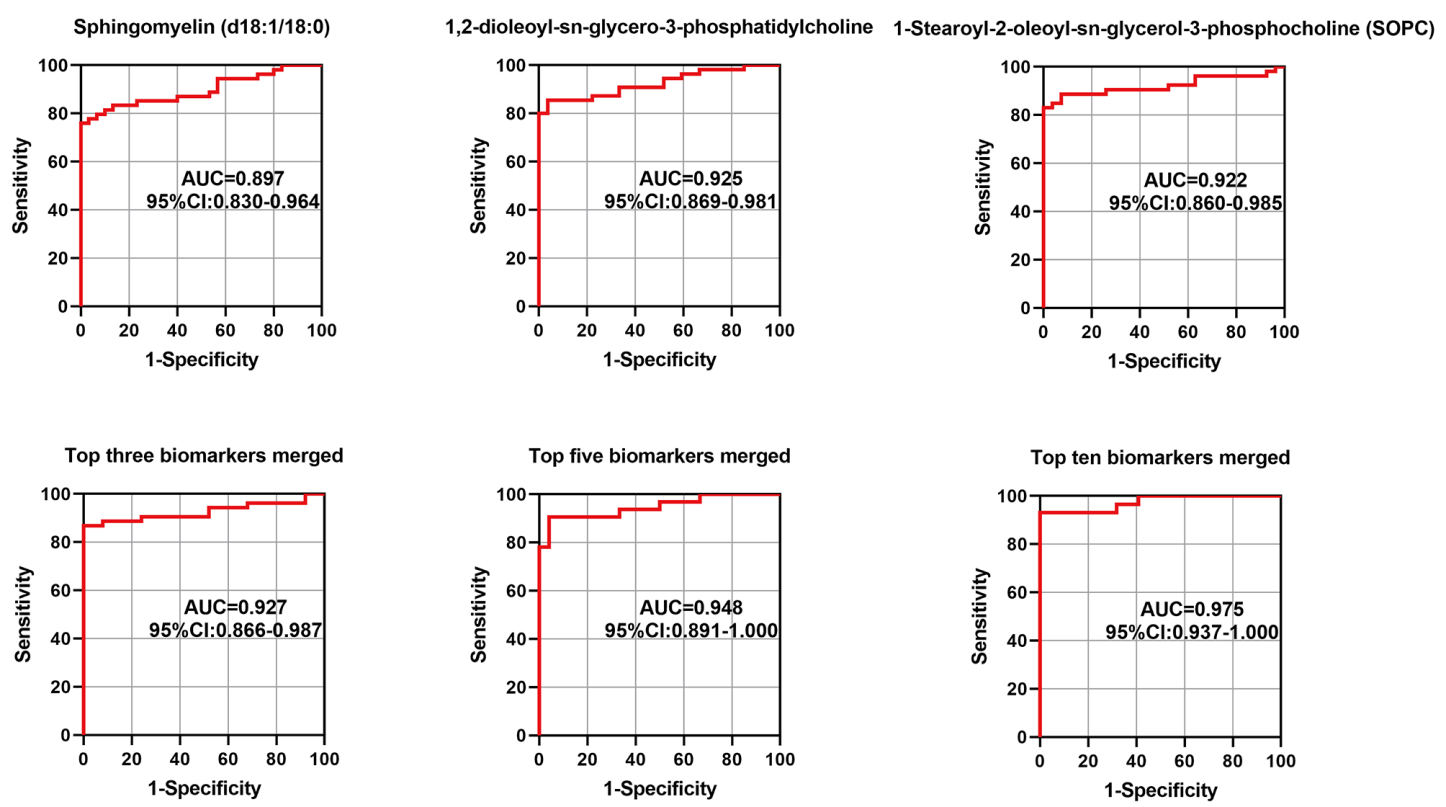
Saliva ROC curve of Sphingomyelin (d18:1/18:0), 1,2-dioleoyl-sn-glycero-3-phosphatidylcholine, 1-Stearoyl-2-oleoyl-sn-glycerol 3-phosphocholine (SOPC) and merged biomarker

ROC analysis of potential biomarkers was performed to identify metabolites with the capability of diagnosing CEG. For the ROC curve analysis, 0.5 < AUC ≤ 0.7, 0.7 < AUC ≤ 0.9 and 0.9 < AUC < 1.0 indicate low, medium and high diagnostic accuracy, respectively. ROC analysis was performed based on the FC values of the top ten metabolites, and the cumulative AUC of the top three and top ten metabolites was calculated (Table 2; Fig. 6). The top three metabolites, such as sphingomyelin (d18:1/18:0), 1,2-dioleoyl-sn-glycero-3-phosphatidylcholine, and 1-stearoyl-2-oleoyl-sn-glycerol-3-phosphocholine(SOPC), demonstrated the AUC values of 0.897 (95%CI: 0.830–0.963), 0.925 (95%CI: 0.869–0.981) and 0.922 (95%CI: 0.860–0.985), respectively. Moreover, the cumulative AUC values of the top three, five and ten biomarkers were 0.927 (95%CI: 0.866–0.987), 0.948 (95%CI: 0.891-1.000) and 0.975 (95%CI: 0.937-1.000), respectively. These results implied a high accuracy of metabolite biomarkers in predicting the occurrence of CEG.

**Table 2:** ROC analysis of CEG top 10 biomarkers from saliva

Metabolites	Cut.offs	Sensitivity	Specificity	AUC	p value
Sphingomyelin (d18:1/18:0)	7100.78	0.759	1.000	0.897	1.9426E-9
1,2-dioleoyl-sn-glycero-3-phosphatidylcholine	3906.03	0.855	0.963	0.925	4.934E-10
1-Stearoyl-2-oleoyl-sn-glycerol-3-phosphocholine (SOPC)	13346.74	0.830	1.000	0.922	7.7141E-10
Lys-Pro	10460.62	0.711	0.750	0.781	0.000105
1-Palmitoyl-sn-glycero-3-phosphocholine	5267.02	0.673	0.767	0.778	0.000038
N-Acetylcadaverine	10026.48	0.702	0.962	0.867	2.312E-7
1-Stearoyl-2-hydroxy-sn-glycero-3-phosphocholine	5135.58	0.654	0.867	0.762	0.000086
Indoleacetic acid	7754.61	0.649	0.962	0.843	6.1138E-7
(3-Carboxypropyl)trimethylammonium cation	52372.3	0.768	0.885	0.863	1.4293E-7
Thioetheramide-PC	103381.19	0.725	1.000	0.875	1.2218E-7

### Bioinformatics analysis of the metabolite biomarkers

**Fig. 7:**
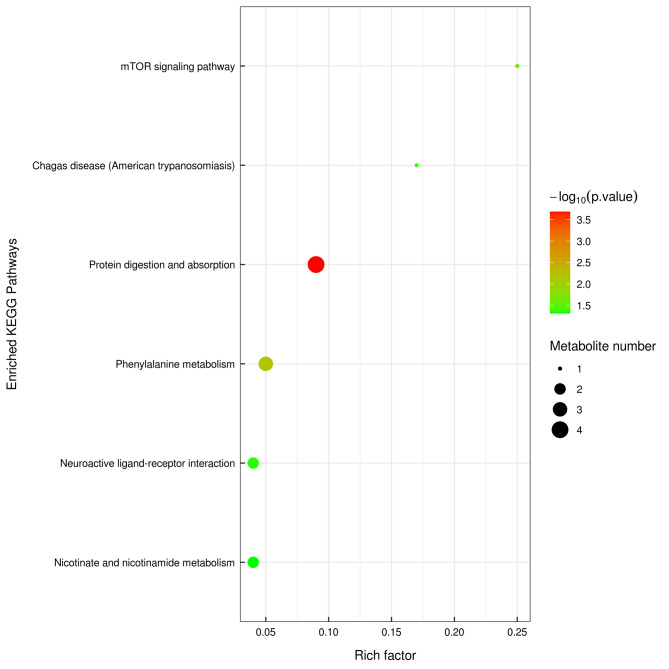
Kyoto encyclopedia of genes and genomes (KEGG) pathway enrichment analysis of differentially expressed metabolites

The differential metabolites screened between the CEG and normal groups were mainly classified as organic acids and derivatives, lipids and lipid-like molecules, organoheterocyclic compounds, organic oxygen compounds, and benzenoids. KEGG pathway analysis was performed to further explore the most relevant metabolic pathways. In order to determine which metabolic and signal transduction pathways were significantly affected, the significance level of metabolite enrichment in each pathway was analyzed. The 6 significantly affected signal transduction pathways are described in Fig. 7, which include protein digestion and absorption, phenylalanine metabolism, neuroactive ligand-receptor interaction, nicotinate and nicotinamide metabolism, mTOR signaling pathway.

## Discussion

As a long-term chronic inflammation of gastric mucosa, CEG is closely related to gastric cancer [25]. Therefore, early diagnosis and intervention of CEG are of great significance. At present, the diagnosis of CEG mainly depends on the examination of electronic gastroscope, but the characteristics of invasiveness and discomfort have limited its widespread applications. Consequently, a simple and safe detection method is highly required. Saliva is one of the most important human body fluids, which is easy to obtain and has great potential in disease diagnosis. The aim of this study was to identify potential biomarkers from the saliva of CEG patients.

In this study, the baseline data of sex, age, ALT, AST and ALP were evenly matched between the two groups, and there was no significant deviation. CEG patients showed epigastric pain, gastric distension and heartburn, but there was no significant difference in the incidence of the three symptoms. This is consistent with previous understanding. CEG patients can have a variety of common clinical symptoms of the upper digestive system, but these symptoms are not specific and cannot identify the occurrence of this disease [26].

Metabonomics technology was used to verify the metabolic differences between CEG patients and healthy controls. A total of 45 metabolites were identified and annotated as potential biomarkers, and 6 metabolic pathways were enriched. Notably, the levels of L-arginine, tyramine, indoleacetic acid, phenylpyruvate and N-acetylputrescine were all elevated in the saliva of CEG patients. L-arginine is an essential amino acid, which plays important roles in regulating physiological and biochemical processes. It has been reported that L-arginine can aggravate the damage of ethanol to rat gastric mucosa [27]. L-arginine is a donor of nitric oxide, which can promote gastric ulcer in gastric ischemia-reperfusion [28]. A previous study found that the metabolism of L-arginine in patients with advanced gastric adenocarcinoma was more active than that in patients with superficial gastritis [29], which was consistent with the results of this experiment. The high metabolic level of L-arginine may be the factor contributing to CEG, suggesting that CEG is at risk of further deterioration. Tyramine is a kind of biological trace amine, which is produced by tyrosine deacidification, and diet is its main source [30]. Tyramine can be used as an agonist of human trace amine-associated receptor to stimulate G cells in pyloric tissue for gastrin production, promote the secretion of gastric juice, and alter the movement state of stomach [31]. In this study, the salivary level of tyramine in CEG patients was higher than that of healthy controls. The effect of tyramine may increase gastric acid secretion, cause high acid state in the stomach, damage gastric mucosa, and delay its repair.

In this study, we found that the levels of 6 substances related to lipid metabolism had changed significantly. The levels of arachidic acid and behenic acid were decreased in the saliva of CEG patients, and both of them were involved in the biosynthesis of unsaturated fatty acids. Previous studies showed that unsaturated fatty acids could reduce oxidative damage and inflammatory response [32], enhance the defense of gastric mucosa [33], promote the repair of gastric mucosa [34], and reduce peptic ulcer [35]. Similar to the results of this study, the serum levels of six unsaturated free fatty acids in patients with gastric cancer were significantly lower than those of patients with benign gastric disease [36]. Polyunsaturated fatty acids exhibited tumoricidal action on gastric cancer cells in vitro [37], and had been proposed as adjuvant treatment in cancer due to their excellent anti-inflammatory properties [38]. These results suggest that CEG is related to lipid metabolism.

Through the metabolic pathway enrichment analysis, mTOR signaling pathway was found to be associated with CEG. mTOR signaling pathway was considered to be a key regulator of autophagy [39]. Autophagy plays an important role in maintaining cell homeostasis, and is closely related to the occurrence of many human diseases, including cancer [40]. In previous studies [41, 42], inhibition of autophagy can lead to gastric mucosal epithelial cell apoptosis and gastric mucosa damage. Activation of autophagy by downregulating mTOR signaling pathway can ameliorate ethanol-induced gastric mucosal epithelial cell injury. More importantly, a measure of autophagy can induce gastric cancer cell apoptosis, inhibit gastric cancer cell proliferation, and increase their sensitivity to chemotherapeutic drugs [43]. In this study, the level of L-arginine in CEG patients was higher than that in healthy controls. Arginine is an amino acid critically involved in multiple cellular processes, and is a direct activator of mTOR [44]. Therefore, we speculate that L-arginine activates mTOR signaling pathway to regulate the autophagy of gastric mucosal epithelial cells, which may be one of the mechanisms leading to CEG.

However, the present study has some limitations. First, to improve the reliability of CEG diagnosis, a larger sample size is need for designing the test set and validation set. Second, in this study, the samples before CEG treatment were selected, and the treated samples were not selected. Therefore, the treated samples can be added to the future work to verify the results of this study, as well as explore new treatment methods and targets. In the following work, targeted metabolomics techniques will be used to further analyze metabolites in saliva of CEG patients. Groups based on gender, age and other factors will also be incorporated into subsequent studies.

## Conclusion

In summary, 45 potential biomarkers related to CEG were identified and 6 metabolic pathways were enriched. These differential metabolites were related to amino acid, lipid, phenylalanine metabolism, protein digestion and absorption, and mTOR signaling pathway. In addition, we found that 1,2-dioleoyl-sn-glycoro-3-phosphocholine and 1-stearoyl-2-oleoyl-sn-glycoro-3-phospholine (SOPC) had the potential to become metabolic biomarkers for diagnosing CEG. The results of this study are helpful to identify potential biomarkers for non-metabolic diseases.

## Electronic supplementary material

Below is the link to the electronic supplementary material.

Supplementary Material 1

Supplementary Material 2

Supplementary Material 3

Supplementary Material 4

Supplementary Material 5

## Data Availability

The data that support the findings of this study is available from the corresponding author upon reasonable request.
