# Supplementary figures and images for: Changes in the salivary metabolome in patients with chronic erosive gastritis
Source: BMC Gastroenterol. 2023 May 19;23:161. doi: 10.1186/s12876-023-02803-6 (PMC10197399; doi:10.1186/s12876-023-02803-6)

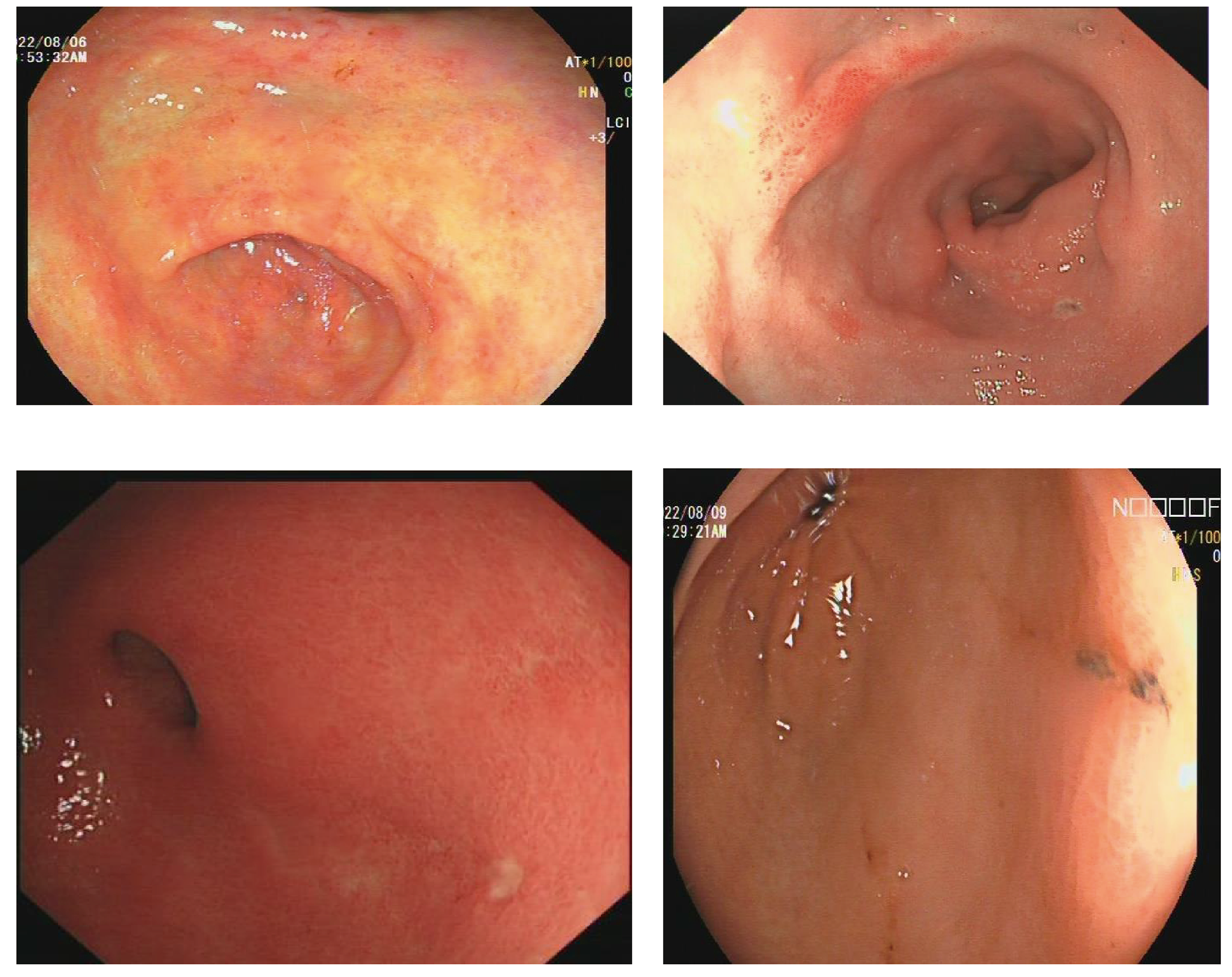

Supplement: Supplementary file 1 — Supplementary Material 1 [file 12876_2023_2803_MOESM1_ESM.png]

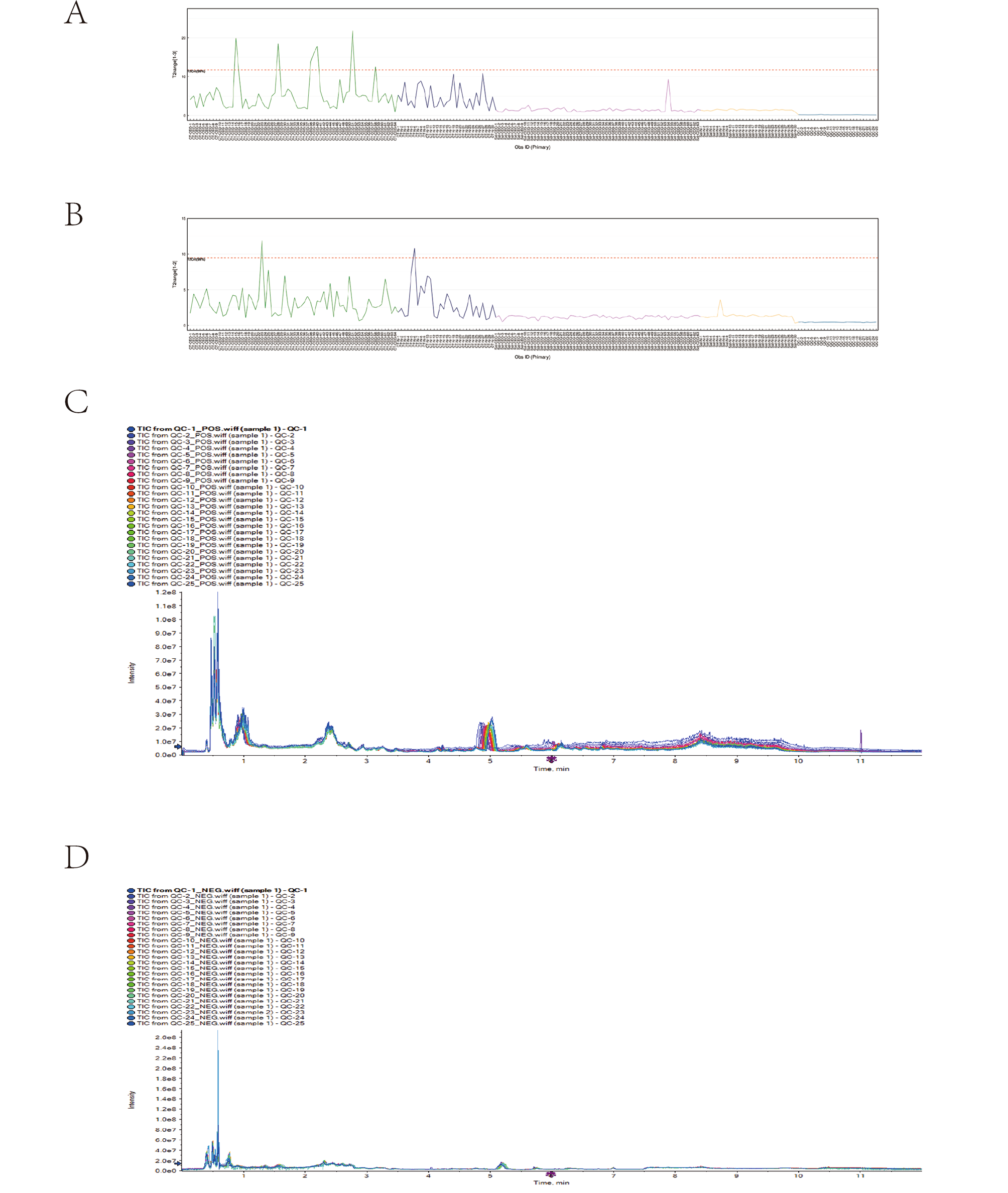

Supplement: Supplementary file 2 — Supplementary Material 2 [file 12876_2023_2803_MOESM2_ESM.png]

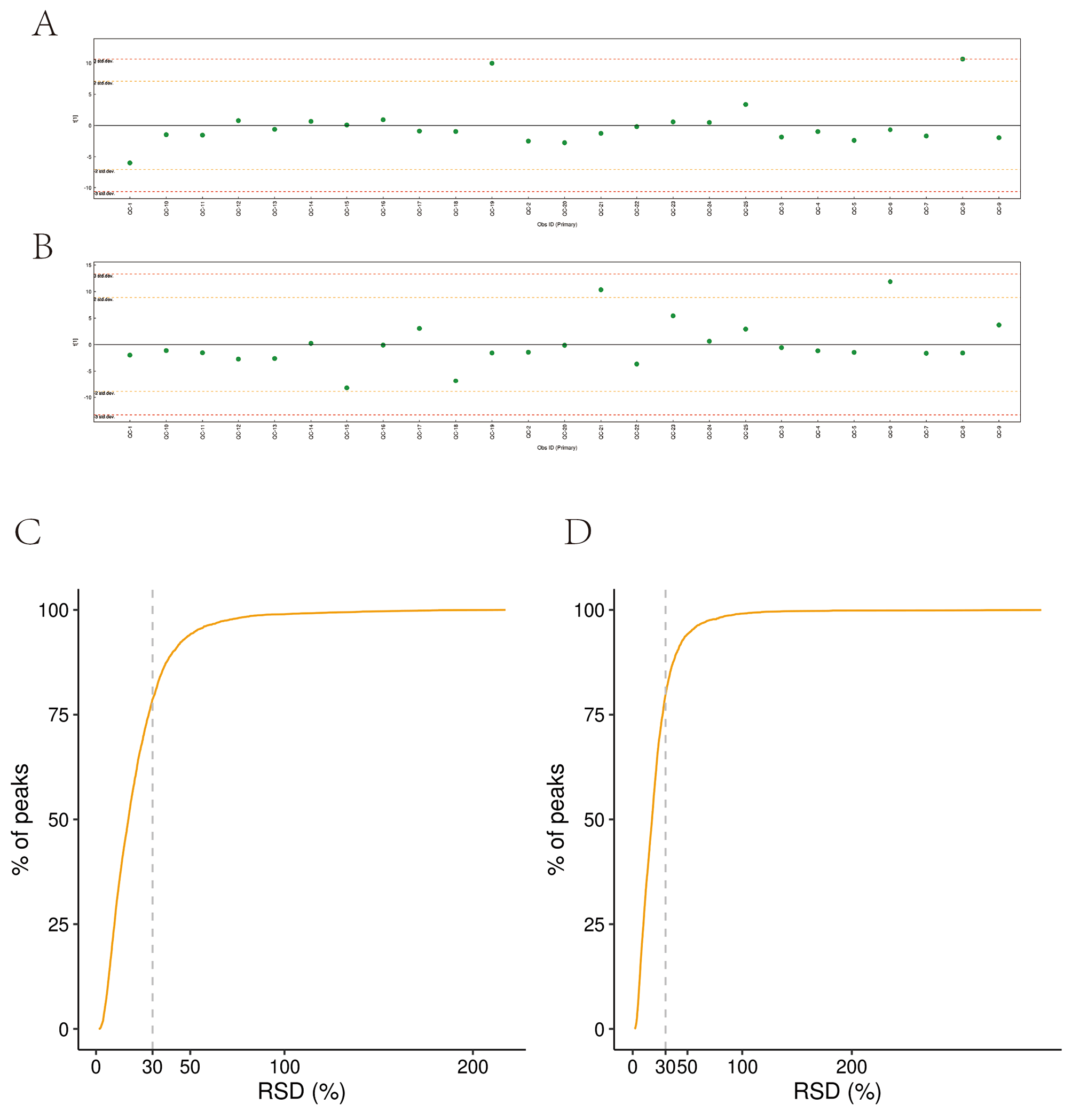

Supplement: Supplementary file 3 — Supplementary Material 3 [file 12876_2023_2803_MOESM3_ESM.png]
