# Supplementary material for: Changes in the salivary metabolome in patients with chronic erosive gastritis
Source: BMC Gastroenterol. 2023 May 19;23:161. doi: 10.1186/s12876-023-02803-6 (PMC10197399; doi:10.1186/s12876-023-02803-6)
Supplement: Supplementary file 4 — Supplementary Material 4 [file 12876_2023_2803_MOESM4_ESM.docx]

Supplemental Table 1 Potential biomarkers of saliva samples from healthy and CEG subjects

| **Adduct** | **Detection Model** | **Name** | **VIP** | **Fold change** | ***p*-value** | **FDR** | **m/z** | **rt(s)** |
| --- | --- | --- | --- | --- | --- | --- | --- | --- |
| (M+CH3CN+H)+ | HILIC(+) | Sphingomyelin (d18:1/18:0) | 2.08 | 20.42 | ＜0.001 | 0.001 | 772.61521 | 141.3305 |
| (M+H-H2O)+ | HILIC(+) | 1,2-dioleoyl-sn-glycero-3-phosphatidylcholine | 1.70 | 14.02 | ＜0.001 | ＜0.001 | 768.58416 | 139.4255 |
| (M+H)+ | HILIC(+) | 1-Stearoyl-2-oleoyl-sn-glycerol 3-phosphocholine (SOPC) | 2.95 | 13.99 | ＜0.001 | 0.001 | 788.61056 | 141.995 |
| (M+H)+ | HILIC(+) | Lys-Pro | 2.51 | 10.05 | 0.024 | 0.024 | 244.16428 | 413.274 |
| (M+H)+ | HILIC(+) | 1-Palmitoyl-sn-glycero-3-phosphocholine | 1.29 | 6.93 | 0.001 | 0.001 | 496.33723 | 190.515 |
| (M+H)+ | HILIC(+) | N-Acetylcadaverine | 1.97 | 6.35 | ＜0.001 | 0.001 | 145.13245 | 288.9405 |
| (M+H)+ | HILIC(+) | 1-Stearoyl-2-hydroxy-sn-glycero-3-phosphocholine | 1.13 | 5.54 | ＜0.001 | 0.001 | 524.36759 | 186.9875 |
| (M-H)- | HILIC(-) | Indoleacetic acid | 1.09 | 4.94 | 0.012 | 0.014 | 174.05443 | 133.957 |
| M+ | HILIC(+) | (3-Carboxypropyl)trimethylammonium cation | 4.39 | 4.82 | ＜0.001 | 0.001 | 146.11673 | 368.6015 |
| (M+Na)+ | HILIC(+) | Thioetheramide-PC | 4.69 | 4.76 | ＜0.001 | ＜0.001 | 758.5667 | 143.146 |
| (M+H)+ | HILIC(+) | 1-Oleoyl-sn-glycero-3-phosphocholine | 1.10 | 4.67 | ＜0.001 | 0.001 | 522.35243 | 182.7145 |
| (M-H)- | HILIC(-) | 3,4-Dihydroxybenzoate (Protocatechuic acid) | 1.21 | 4.30 | 0.013 | 0.015 | 153.01821 | 49.986 |
| (M+H)+ | HILIC(+) | Pro-Arg | 4.42 | 3.91 | 0.016 | 0.018 | 272.17044 | 442.422 |
| (M-H)- | HILIC(-) | Propionic acid | 1.10 | 3.86 | 0.008 | 0.010 | 73.02933 | 157.718 |
| (M+H-H2O)+ | HILIC(+) | Glu-Pro | 1.84 | 3.65 | 0.013 | 0.015 | 227.10154 | 359.0385 |
| (M+H)+ | HILIC(+) | N-Acetylputrescine | 1.51 | 3.44 | 0.003 | 0.004 | 131.11728 | 313.017 |
| (M-H)- | HILIC(-) | DL-3-Phenyllactic acid | 4.27 | 3.39 | 0.001 | 0.002 | 165.05449 | 113.8885 |
| (M+H)+ | HILIC(+) | gamma-L-Glutamyl-L-glutamic acid | 1.06 | 3.19 | 0.002 | 0.003 | 277.10174 | 452.922 |
| (M+H-H2O)+ | HILIC(+) | Tyramine | 2.11 | 3.08 | 0.003 | 0.005 | 120.07997 | 254.308 |
| (M-H)- | HILIC(-) | Hydroxyisocaproic acid | 3.11 | 3.01 | 0.016 | 0.017 | 131.07088 | 126.065 |
| (M+H)+ | HILIC(+) | 3,3-Dimethylacrylic acid | 2.44 | 2.97 | ＜0.001 | ＜0.001 | 101.05879 | 375.822 |
| (M+H)+ | HILIC(+) | .beta.-Homoproline | 1.27 | 2.95 | 0.008 | 0.009 | 130.08514 | 319.411 |
| (M-H)- | HILIC(-) | 3-Phenylpropanoic acid | 14.47 | 2.89 | 0.001 | 0.002 | 149.06011 | 94.123 |
| (M-H)- | HILIC(-) | Taurine | 4.24 | 2.72 | ＜0.001 | 0.001 | 124.00675 | 286.509 |
| (M+H)+ | HILIC(+) | His-Glu | 1.46 | 2.68 | 0.002 | 0.003 | 285.11756 | 412.4475 |
| (M-H)- | HILIC(-) | Phenylpyruvate | 1.11 | 2.55 | ＜0.001 | ＜0.001 | 163.03851 | 51.315 |
| (M-H)- | HILIC(-) | 3-Indolepropionic acid | 1.30 | 2.46 | 0.003 | 0.006 | 188.06985 | 99.643 |
| (M+H)+ | HILIC(+) | Arg-Glu | 1.46 | 2.41 | 0.001 | 0.002 | 304.15971 | 445.635 |
| (M+H)+ | HILIC(+) | Thr-Phe | 1.60 | 2.40 | 0.002 | 0.004 | 267.13242 | 222.645 |
| (M-H)- | HILIC(-) | Isobutyric acid | 2.53 | 2.20 | 0.001 | 0.002 | 87.04496 | 100.857 |
| (M+H)+ | HILIC(+) | Arg-Ala | 1.36 | 2.14 | 0.011 | 0.013 | 246.15445 | 407.3875 |
| (M+H)+ | HILIC(+) | N1-Methyl-2-pyridone-5-carboxamide | 2.74 | 2.08 | 0.004 | 0.005 | 153.06495 | 76.988 |
| (M-H)- | HILIC(-) | Valeric acid | 3.68 | 1.91 | 0.001 | 0.003 | 101.06027 | 91.341 |
| (M-H)- | HILIC(-) | Isocaproic acid | 4.25 | 1.59 | 0.029 | 0.029 | 115.07572 | 75.165 |
| (M+H)+ | HILIC(+) | L-Arginine | 3.76 | 1.41 | 0.042 | 0.042 | 175.11787 | 526.7585 |
| (M+H-H2O)+ | HILIC(+) | Phenylacetic acid | 1.17 | 1.32 | ＜0.001 | ＜0.001 | 119.04802 | 32.894 |
| (M+H-H2O)+ | HILIC(+) | 1-Stearoyl-rac-glycerol | 1.76 | 1.15 | 0.005 | 0.007 | 341.30289 | 32.4895 |
| (M-H)- | HILIC(-) | Behenic acid | 1.18 | 0.80 | 0.004 | 0.006 | 339.32205 | 40.566 |
| (M-H)- | HILIC(-) | Pinocembrin | 1.08 | 0.75 | 0.005 | 0.007 | 255.06655 | 28.358 |
| (M-H)- | HILIC(-) | Arachidic acid | 2.22 | 0.72 | ＜0.001 | ＜0.001 | 311.2919 | 41.404 |
| (M+CH3COO+2H)+ | HILIC(+) | 2-Ethoxyethanol | 1.10 | 0.71 | ＜0.001 | ＜0.001 | 151.0953 | 25.789 |
| (M+H)+ | HILIC(+) | Dioctyl phthalate | 1.23 | 0.65 | 0.006 | 0.007 | 391.28134 | 32.9365 |
| (M-H)- | HILIC(-) | 2-Methylbenzoic acid | 3.93 | 0.55 | ＜0.001 | ＜0.001 | 135.04413 | 101.181 |
| (M-H)- | HILIC(-) | Norethindrone Acetate | 11.48 | 0.50 | ＜0.001 | ＜0.001 | 339.19619 | 26.3465 |
| (M-H)- | HILIC(-) | Tetrahydrocorticosterone | 1.08 | 0.44 | ＜0.001 | ＜0.001 | 349.23654 | 23.444 |
